# Physical and psychosocial work environment factors and their association with health outcomes in Danish ambulance personnel – a cross-sectional study

**DOI:** 10.1186/1471-2458-12-534

**Published:** 2012-07-23

**Authors:** Claus D Hansen, Kurt Rasmussen, Morten Kyed, Kent Jacob Nielsen, Johan Hviid Andersen

**Affiliations:** 1 Department of Sociology and Social Work, Aalborg University, Aalborg University, Kroghstræde 5, DK 9220, Aalborg Ø, Denmark; 2 Danish Ramazzini Centre, Department of Occupational Medicine, Regional Hospital Herning, Gl. Landevej 61, DK-7400, Herning, Denmark

## Abstract

**Background:**

Reviews of the literature on the health and work environment of ambulance personnel have indicated an increased risk of work-related health problems in this occupation. The aim of this study was to compare health status and exposure to different work environmental factors among ambulance personnel and the core work force in Denmark. In addition, to examine the association between physical and psychosocial work environment factors and different measures of health among ambulance personnel.

**Methods:**

Data were taken from a nationwide sample of ambulance personnel and fire fighters (n = 1,691) and was compared to reference samples of the Danish work force. The questionnaire contained measures of physical and psychosocial work environment as well as measures of musculoskeletal pain, mental health, self-rated health and sleep quality.

**Results:**

Ambulance personnel have half the prevalence of poor self-rated health compared to the core work force (5% vs. 10%). Levels of mental health were the same across the two samples whereas a substantially higher proportion of the ambulance personnel reported musculoskeletal pain (42% vs. 29%). The ambulance personnel had higher levels of emotional demands and meaningfulness of and commitment to work, and substantially lower levels of quantitative demands and influence at work. Only one out of ten aspects of physical work environment was consistently associated with higher levels of musculoskeletal pain. Emotional demands was the only psychosocial work factor that was associated with both poorer mental health and worse sleep quality.

**Conclusions:**

Ambulance personnel have similar levels of mental health but substantially higher levels of musculoskeletal pain than the work force in general. They are more exposed to emotional demands and these demands are associated with higher levels of poor mental health and poor sleep quality. To improve work environment, attention should be paid to musculoskeletal problems and the presence of positive organizational support mechanisms that can prevent negative effects from the high levels of emotional demands.

## Background

Systematic reviews of the literature on the health and work environment of ambulance personnel have over the last decade emphasised what appears to be an increased risk of developing work-related health problems among this particular occupational group [1-3]. Earlier studies have shown that ambulance personnel have a ten-fold higher rate of early retirement than nurses, and a doubled risk compared to those carrying out manual work in the health sector. The leading causes of early retirement are musculoskeletal disorders (MSD), diseases of the circulatory system and mental illness [4,5]. Of special interest has been the observation that emergency service work is inherently stressful. Ambulance personnel need to provide medical assistance in critical and unknown situations where those in need of help are at risk of dying if care is not given swiftly and appropriately. In addition, ambulance personnel will often face situations in which relatives afraid of losing their loved ones or other bystanders will be watching them while they carry out their work. All of this contributes to the inherently stressful nature of their job [6].

Because of the stressful nature of emergency service work, most studies of ambulance personnel have addressed psychological health outcomes [7-13]. Posttraumatic stress disorder (PTSD) symptoms have been reported with prevalences of 15–20% among ambulance personnel, which is 4 to 10 times higher than in the general population and far above what is seen in other occupational groups exposed to sudden serious psychological hazards [7,8]. Furthermore, other psychological outcomes such as burnout, depression and anxiety have been the topic of a few studies, where an elevated prevalence of symptoms has been reported [9-13]. Unfortunately, most studies lack the data quality or normative data that would enable comparisons across different occupations. Thus, overall there is a lack of conclusive evidence about the prevalence of mental illness and other psychological outcomes among ambulance personnel compared to other occupations on the labour market (1). The same is seen in studies of the somatic health of ambulance personnel, where even fewer studies were identified in recent reviews of the literature [1,2]. MSDs have only been investigated in regard to well-known hazards such as heavy lifting, bending and carrying [14,15]. However, other physical exposures are probably also relevant because of the unpredictable nature of emergency service work. Exposures such as working in awkward postures with sudden and unexpected movements or having to perform maximum force exertions should be investigated as well. Working with in-transit care in the rescue vehicle, which requires reaching for overhead equipment and horizontal bending and twisting, has been identified as the most risky exposure for developing musculoskeletal pain in an American study based on observations of training exercises [16]. All in all, this also points to a lack of research into important aspects of the physical work environment of ambulance personnel.

### Ambulance services in Denmark

In Denmark, five regional counties have the responsibility for hospital and pre-hospital service. Contrary to several other countries, no hospitals in Denmark have their own integrated ambulance departments. Since 1963, there has only been one major ambulance service provider in Denmark. This private provider has covered most of the country apart from areas within four counties where the fire department also runs the ambulance service (covering Copenhagen, the Danish capital, and some counties in the vicinity in which another private contractor has won the tender). Besides ambulance service, the company also performs the majority of other emergency services in Denmark, including fire fighting, animal rescue, patient transportation, roadside assistance, environmental assistance, and various forms of health care education and assistance to private people and public institutions. In 2008, Denmark’s five county governments have started putting ambulance service out to tender every fourth year. The first tender changed little in the overall structure of the Danish ambulance service, with one new private ambulance provider taking over some of the districts that had formerly been run by the original company. Despite this, the biggest ambulance provider still covers 85% of all emergency operations in Denmark. All in all, approximately 4500 persons are employed as ambulance personnel in Denmark. Each county assembles the pre-hospital service individually and puts the service to tender. The set-up and the running of the pre-hospital service differ slightly between the five Danish counties. Some counties have, for example, chosen a model with a specific permanent emergency standby, defining the numbers of ambulances, personnel, ambulance stations, etc. In other counties, there is no minimum permanent emergency standby. The ambulance service provider instead has the responsibility for ensuring specific maximum response times, but has considerable autonomy to set up the emergency standby as it finds it most appropriate to ensure the contractually required response times.

Until the middle of the 1990s, Danish ambulance workers received little formal education. However, as the ambulances became increasingly equipped, and especially with the introduction of the defibrillator, a formal emergency worker/ambulance assistant (level 1) vocational education was introduced. Subsequently, an additional 5 week treatment provider (level 2) education was introduced, and in 2004, a formal 11 week paramedic (level 3) education was introduced. Hence, unlike many countries, Danish ambulances are not staffed by nurses. Danish legislation requires that ambulances must be staffed with at least two persons. At least one of these must be an educated treatment provider and the other must have passed an exam as an assistant provider. Most of the ambulance personnel perform more than one job function, e.g., rotating between emergency ambulance driving and transportation of patients over the work week. However, procurement rules require that companies that compete for tender must keep their sectional services separate in the daily operation. This has introduced a more rigid handling of jobs that could affect the work environment. The former board portfolio of jobs gave the local station-manager more flexibility to spare ambulance-workers by assigning them to less demanding tasks such as patient transportation for smaller or longer periods of time.

### Aims and scopes of the paper

The aim of this paper is threefold: 1) to compare various aspects of health status among Danish ambulance personnel with that of the general work force in order to establish whether prior findings of more musculoskeletal pain and more mental health problems in this occupation can be replicated; 2) to describe the general work environment among ambulance personnel and compare the psychosocial work environment to other occupations; and 3) to examine the associations between the physical and psychosocial work environments and five health outcomes.

## Methods

### Participants

The data used in this paper stem from the first round of the cohort-study MARS (Men, Accidents, Risk & Safety), whose primary aim is to examine the impact of gender identity on risk-taking behaviour, attitudes towards safety, and occupational accidents. Data were collected on standard work environment factors and self-rated general health in order to shed light on some of the lesser known aspects of the work environment exposures in emergency service work. The participants in this study come from the biggest ambulance service provider in Denmark, covering 85% of all emergency calls.

In total, 3,888 employees (i.e., all full-time employees in the company’s emergency services) covering 127 distinct emergency service stations were invited to participate in the study in October 2010. The participants had a choice to fill out the questionnaire either online or using a traditional post-distributed questionnaire. After 5 rounds of invitations and reminders a total of 2,426 completed the questionnaire fully or in part, yielding a response rate of 62.4%. Of these, 54.9% (1,332) used the online questionnaire and the remainder filled out the paper-based questionnaire.

Examining the characteristics of the non-responders showed that the response rate among women was somewhat lower (56% vs. 63%), and that the older employees were more likely to participate compared to the youngest group. Finally, there were substantial differences across the 66 emergency service stations having more than 15 employees, with response rates ranging from 40.4% to 84.3%. Grouping the stations by geographical region revealed that there were slightly lower response rates in the capital area compared to some of the more rural parts of the country, but the differences were not that great (56% vs. 66%). All in all, the sample is appropriate to capture any differences between emergency departments located in urban and rural parts of the country and the non-responders were not substantially different from those who chose to participate.

**Figure 1:**
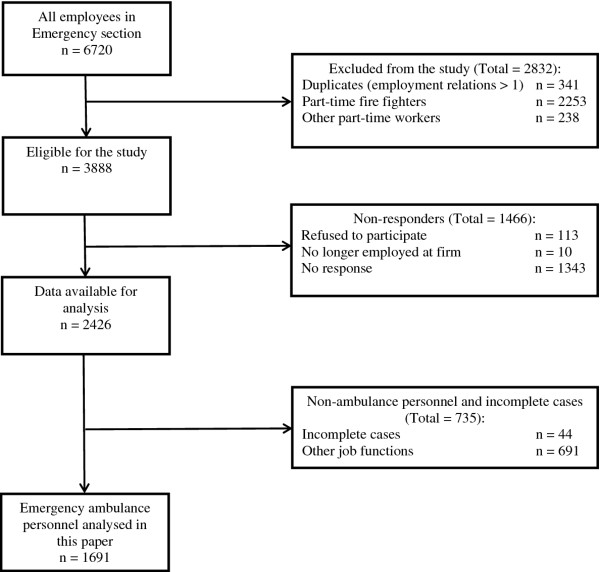
Flowchart of the study population.

We asked the participants to indicate all the job functions they were currently carrying out. Additionally, we asked them to specify which of these functions occupied most of their working time. In this analysis, we included those indicating that they worked as emergency ambulance personnel (n = 1,691). Figure 1 is a flowchart of the study population and sample used in this paper. The study was approved by the Danish Data Protection Agency (Study No. 2010-41-4817).

### Reference samples

Data from a large national survey (n = 14,241) conducted in September 2004 were used to make comparisons of the health status between ambulance personnel and the core work force in Denmark. The national survey contained the same health measures as the current study with only minor differences in response categories and wording. In the current study, participants were asked to indicate the level of pain in neck, shoulders and arms in one single measure, whereas this was asked in two items in the reference sample. The two items in the reference sample were then added up and the mean was taken in order to be able to compare it across samples. Similarly, the two items from the mental health dimension of SF-12 had 6 response categories in the current study and 5 in the reference sample. We collapsed two of the response categories in the current study and recoded the items to a single dichotomous variable indicating low level of mental health. The measures of low back pain and self-rated general health were identical in the two samples. Unfortunately, the reference sample did not include questions on sleep quality, disallowing us from comparing this health measure across samples.

No samples were identified that would enable us to compare the physical work environment factors in the current study with the work force in general. Instead, a subsample of all those indicating fire fighting as a job function (n = 397) working within the same emergency service provider was used as an internal comparison group.

The developers of the COPSOQ (Copenhagen Psychosocial Questionnaire) provided reference material for the psychosocial work environment factors [17].

### Outcome measures

Five different measures of health were used to assess the health status of the ambulance personnel. Self-rated general health was measured using the single item global measure taken from SF-36 [18]. This variable was dichotomised to capture those reporting poor self-rated general health. For use in the multivariate analysis the dichotomisation was changed as follows (excellent, very good = 1) (good, not so good, poor = 1) because of the low number of respondents indicating not so good and poor self-rated health (n = 59). Sleep quality was measured using a single global item taken from the Basic Nordic Sleep Questionnaire [19]. This variable was dichotomised to capture those reporting bad sleep qualities. Musculoskeletal pain in neck, shoulder and arms as well as low back pain was measured using two global measures tested and used in several other Danish studies [20,21]. These variables were dichotomised to indicate moderate to high levels of pain. Finally, the two-item mental health scale from SF-12 was used to assess any mental health problems among the participants (Cronbach’s α = 0.6). This variable was also dichotomised to indicate those who had poorer mental health than the average in the Danish population.

### Physical work environment

10 items from Dutch Musculoskeletal Questionnaire (DMQ) [22] were used to assess some of the most important physical work environment exposures for the ambulance personnel. Instead of using the original yes vs. no response categories, they were altered in order to assess how often during a day each of the 10 exposures were experienced (‘never’, ‘seldom’, ‘once or twice daily’, ‘3–10 times a day’, ‘more than 10 times a day’). For use in analyses, the items were dichotomised to indicate daily exposure to these situations. Analyses, the items were used as continuous variables.

### Psychosocial work environment

The short version of the Copenhagen Psychosocial Questionnaire (COPSOQ) was used to assess the psychosocial work environment among the ambulance personnel [23]. This version contained two items for each of the 11 subscales. Cronbach’s α ranged from 0.3 to 0.7 for the scales. The items were added, yielding variables ranging from 0 to 8 for each of the subscales. The items were dichotomised using the means from the reference sample as cut-off points to indicate high vs. low levels of exposure to these factors. 

### Confounders

For use in the descriptive analyses and as potential confounder controls in the multivariate analyses, the following variables were included: age, sex, marital status, body mass index, alcohol consumption and level of physical activity. The potential confounders were selected because they could be associated with both the outcome and the work environment exposures, as in the case of age, sex, body mass index and physical activity where this is most likely with physical work environment and musculoskeletal pain. Marital status and alcohol consumption were included as confounders because they have been found to be associated with mental health and could also have an impact on how the respondents rate their psychosocial work environment. In order for the analyses to be consistent, the complete set of confounders was used for all five outcomes.

### Statistics

To compare the health status and work environment of the ambulance personnel with the fire fighters and the reference samples, simple Student’s T-tests of independence and 95% Confidence Intervals were calculated. In order to examine the association between the physical and psychosocial work environment and the five different measures of health status, multiple logistic regressions were carried out. The variables were entered in two steps into two models. First, all physical and psychosocial work environment factors were entered into the regression model one at a time adjusting only for the set of confounders (Model 1). Only physical work environment factors were included as independent variables when musculoskeletal pain was the outcome. For mental health and sleep quality, only psychosocial work environment factors were included as independent variables. For the analysis of self-rated general health, both physical and psychosocial work environment factors were included as independent variables. The second model included all the other physical or psychosocial variables at the same time, still adjusting for the set of confounders. For each of the models, Hosmer Lemeshow Goodness of Fit and Nagelkerke R-Square are reported. In order to discuss the possibility of Type I errors influencing the results, we ran analyses with Benjamini-Krieger-Yekutieli corrected p-values using the smileplot procedure in STATA. All analyses were performed using STATA12 [24].

## Results

Table 1 shows the comparisons of health status between the ambulance personnel and the core work force in Denmark. Half as many of the ambulance personnel rate their health as bad compared to the core work force. This is consistent across age and gender suggesting that ambulance personnel on average perceive themselves as healthier than their colleagues in the work force in general. However, despite this finding, pain in the arms, shoulders and neck as well as low back pain is substantially more prevalent among the ambulance personnel than the work force in general. Nearly half of the ambulance personnel approaching retirement age (60 years in Denmark) experience some degree of musculoskeletal pain compared to just one third of those in the same age group in the core work force. Finally, comparing mental health across occupations, we find that there are no substantial differences—the slight differences for older employees are most likely a consequence of the gender distribution in the two samples. The group of ambulance personnel aged 40–65 consists almost entirely of men, who report better mental health as we know from other studies. The last column in Table 1 shows the prevalence of bad sleep quality in the sample of ambulance personnel only—approximately 20% report having sleeping difficulties and there are only slight differences across age and gender.

**Table 1:** Comparing the prevalence of health problems among ambulance personnel and the core work force in Denmark

	**Pain in arm, shoulder, neck**		**Pain in low back**		**Poor mental health**		**Poor self-rated health**		**Poor quality of sleep**
	**Ambulance personnel (n = 1689)**	**Core work force in Denmark (n = 14175)**	**Ambulance personnel (n = 1690)**	**Core work force in Denmark (n = 14189)**	**Ambulance personnel (n = 1676)**	**Core work force in Denmark (n = 14040)**	**Ambulance personnel (n = 1661)**	**Core work force in Denmark (n = 14097)**	**Ambulance personnel only (n = 1684)**
All	42 (40–44)	29 (29–30)	40 (38–42)	30 (29–31)	18 (16–20)	20 (20–21)	4 (3–4)	11 (10–11)	19 (17–21)
Gender									
Women	44 (34–53)	36 (35–37)	42 (33–52)	33 (32–34)	25 (17–34)	24 (23–25)	1 (0–3)	11 (10–12)	19 (11–26)
Men	42 (40–44)	23 (22–24)	40 (38–42)	27 (26–28)	17 (15–19)	17 (16–18)	4 (3–5)	10 (9–11)	19 (17–21)
Age groups									
18–29	34 (28–39)	24 (22–26)	33 (28–38)	29 (26–31)	21 (17–25)	23 (21–25)	2 (0–3)	7 (6–9)	19 (15–23)
30–39	43 (39–47)	25 (23–26)	42 (38–47)	29 (27–30)	20 (17–24)	21 (20–23)	3 (2–5)	8 (7–9)	19 (16–23)
40–49	44 (39–49)	31 (29–32)	41 (36–46)	30 (29–31)	16 (13–20)	20 (19–21)	4 (2–6)	10 (9–11)	17 (13–21)
50–59	46 (41–51)	35 (34–37)	42 (37–46)	33 (31–34)	13 (10–17)	20 (18–21)	5 (3–7)	15 (14–16)	21 (17–25)
60+	46 (35–58)	25 (22–28)	44 (32–55)	26 (23–29)	13 (5–21)	14 (11–16)	4 (0–9)	9 (7–11)	19 (9–28)

Percentage with 95% Confidence Intervals.

In Table 2, differences in the psychosocial work environment emerge between the ambulance personnel and the work force in general. Due to the large sample sizes, nearly all the differences on the scales measuring different aspects of the psychosocial work environment are statistically significant, the only exception being ‘predictability’. Most notably, ambulance personnel experience much higher levels of emotional demands. Likewise, influence at work is lower among the ambulance personnel. However, the meaning of work and the commitment to their workplace exhibited by the ambulance personnel are substantially higher than that seen in people in the work force in general. In addition, the level of quantitative demands (which is a measure of work load) is substantially lower. In comparing the rest of the dimensions of psychosocial work environment, smaller differences are revealed: possibilities for development are generally better among the ambulance personnel but on the other hand, the levels of vertical trust and the perceived justice and respect of the organization are slightly worse. The most prominent differences, however, are those relating to emotional demands, meaning of work, and influence at work. All of the differences were statistically significant even when applying the Bonferroni correction in order to avoid making Type I errors because of the many hypothesis tests in the table.

**Table 2:** Descriptive statistics of psychosocial work-environment factors among ambulance personnel compared to the core Danish work force

	**Ambulance personnel**	**Workforce in general**	**p-value**
COPSOQ (range: 0–8)			
Quantitative Demands	1.9 (1.2)	3.3 (1.8)	*<0*.*000*
Work Pace	4.3 (1.0)	4.7 (1.6)	*<0*.*000*
Emotional Demands	4.2 (1.3)	3.3 (2.1)	*<0*.*000*
Influence at work	2.9 (1.6)	4.1 (1.8)	*<0*.*000*
Possibilities for development	5.9 (1.2)	5.2 (1.5)	*<0*.*000*
Meaning of work	6.6 (1.1)	6.0 (1.3)	*<0*.*000*
Commitment to workplace	6.0 (1.4)	4.8 (1.8)	*<0*.*000*
Predictability	4.6 (1.4)	4.6 (1.7)	*0*.*698*
Role clarity	6.0 (1.2)	5.7 (1.4)	*<0*.*000*
Vertical trust	5.0 (1.5)	5.4 (1.5)	*<0*.*000*
Justice and respect	4.5 (1.4)	4.8 (1.5)	*<0*.*000*

Mean scores (Standard deviation) and T-tests.

Table 3 compares different aspects of the physical work environment among ambulance personnel and fire fighters working at the same emergency service provider. More than 80% of the ambulance personnel lift heavy loads (>20 kg) at least once each day and more than 60% of the ambulance personnel report daily lifting in an awkward posture or without sufficient surrounding space. As expected, the ambulance personnel had the highest prevalence of all the potential physical exposures on a daily basis, except for slipping or falling during their work, which happened rather seldom for both groups (<5%).

**Table 3:** Descriptive statistics of physical work-environment factors by job functions

**Dutch Musculoskeletal Questionnaire (DMQ) How often do your work imply…**	**Ambulance personnel**	**Fire Fighters**
Lift very heavy loads (more than 20 kg)?	83 (82–85)	59 (54–64)
Lift in an awkward posture	66 (64–69)	51 (46–56)
Not enough room around you to perform your work properly?	65 (62–67)	49 (44–54)
Perform short, but maximal force-exertions	50 (48–53)	41 (36–46)
Lift with a load that is hard to hold	39 (37–41)	32 (26–36)
Make sudden, unexpected movements	39 (36–41)	32 (28–37)
Difficulty in exerting enough force because of incomfortable postures?	37 (35–39)	31 (27–36)
Not enough room above you to perform your work properly?	32 (30–34)	24 (19–28)
Too few facilities to lean on during work?	27 (24–29)	22 (17–26)
Slip or fall during your work?	3 (3–4)	4 (2–6)

Percentage reporting daily exposure with 95% Confidence Intervals.

Table 4 shows the results of the multivariate analyses of musculoskeletal pain and physical work environment. No statistically significant sex differences were found in the study, although the OR for women indicated a slightly higher risk of pain in the neck, shoulder, and arm region. Pain in the neck and shoulder increased with age and this association persisted after adjusting for different work environment factors and the other confounders. Of the physical work environment factors included in our study, only one of the ten included was found to be important for both types of musculoskeletal pain: performing short maximal force exertions increased the odds both of pain in neck, arm, and shoulder and of low back pain. Daily lifting in awkward postures increased the odds of low back pain, but had no effect on pain in the neck whereas lifting a load that is hard to hold increased the odds of pain in neck, arm and shoulder. When calculating the Benjamini-Krieger-Yekutieli corrected p-values, none of the estimates was below the corrected p-values although performing short maximal force exertions was very close.

**Table 4:** Multivariate associations between physical work-environment factors and musculoskeletal pain

			**Pain in neck, arm and shoulder**		**Pain in low back**	
			**Model 1**	**Model 2**	**Model 1**	**Model 2**
**Demographics**						
Sex	Men	Ref	1.00	1.00	1.00	1.00
	Women		1.42 (0.93–2.17)	1.40 (0.90–2.18)	1.47 (0.96–2.25)	1.47 (0.94–2.29)
Age			1.01 (1.00–1.02)	**1.02 (1.01–1.03)**	1.01 (0.99–1.01)	**1.01 (1.00–1.02)**
**Physical work environment factors (daily exposure yes/no)**						
Lift very heavy loads (more than 20 kg)?	No	Ref	1.00	1.00	1.00	1.00
	Yes		1.64 (1.24–2.16)	1.01 (0.74–1.38)	1.61 (1.21–2.12)	1.06 (0.77–1.44)
Lift in an awkward posture?	No	Ref	1.00	1.00	1.00	**1.00**
	Yes		1.91 (1.53–2.38)	1.17 (0.88–1.56)	2.01 (1.61–2.51)	**1.40 (1.04–1.86)**
Not enough room around you to perform your work properly?	No	Ref	1.00	1.00	1.00	1.00
	Yes		1.87 (1.51–2.33)	1.15 (0.86–1.53)	1.68 (1.36–2.09)	1.05 (0.79–1.41)
Perform short, but maximal force-exertions?	No	Ref	1.00	**1.00**	1.00	**1.00**
	Yes		2.04 (1.67–2.50)	**1.41 (1.11–1.80)**	1.79 (1.46–2.19)	**1.29 (1.01–1.64)**
Lift a load that is hard to hold?	No	Ref	1.00	**1.00**	1.00	1.00
	Yes		2.03 (1.65–2.50)	**1.30 (1.00–1.68)**	1.86 (1.51–2.28)	1.26 (0.97–1.62)
Make sudden, unexpected movements?	No	Ref	1.00	1.00	1.00	1.00
	Yes		1.74 (1.42–2.14)	1.13 (0.88–1.47)	1.72 (1.40–2.11)	1.13 (0.87–1.46)
Difficulty in exerting enough force because of uncomfortable postures?	No	Ref	1.00	1.00	1.00	1.00
	Yes		1.92 (1.56–2.36)	1.18 (0.88–1.58)	1.63 (1.32–2.00)	1.01 (0.76–1.36)
Not enough room above you to perform your work properly?	No	Ref	1.00	1.00	1.00	1.00
	Yes		1.50 (1.21–1.86)	0.93 (0.71–1.22)	1.34 (1.09–1.66)	0.90 (0.69–1.17)
Too few facilities to lean on during work?	No	Ref	1.00	1.00	1.00	1.00
	Yes		1.75 (1.40–2.19)	1.12 (0.85–1.48)	1.82 (1.45–2.28)	1.29 (0.98–1.70)
Slip or fall during your work?	No	Ref	1.00	1.00	1.00	1.00
	Yes		2.12 (1.21–3.70)	1.46 (0.80–2.68)	2.38 (1.36–4.15)	1.40 (0.78–2.54)
Hosmer Lemeshow Goodness of fit				Chi^2^ = 1541		Chi^2^ = 1547
				p = 0.424		p = 0.384
Nagelkerke Pseudo R^2^				0.05		0.04
Model 1: Adjusted for sex, age, Body Mass Index, Physical Activity, Marital status & Alcohol consumption.						
Model 2: Model 1 adjusted for all other physical work-environment indicators.						

Odds ratios with 95% Confidence Intervals.

Table 5 contains the results of the multivariate analyses of psychosocial work environment and mental health and sleep quality. First of all, there are sex differences in mental health: women are more likely to report having mental health problems than men, even after adjusting for differences in work environment and the additional confounders. No sex differences were found in sleep quality. Second, higher age is associated with lower odds of reporting mental health problems. Emotional demands seem to be the work environment factor that is most important for the health status of ambulance personnel, as it is associated with both mental health and sleep quality: more emotional demands are associated with poorer mental health and worse sleep quality. Quantitative demands and influence at work are associated with mental health in the expected direction whereas higher levels of justice and respect were associated with lower odds of bad sleep quality. As was the case with the physical work environment factors, none of the estimates had p-values below the Benjamini-Krieger-Yekutieli corrected critical value but the p-value for the association between emotional demands and mental health came very close (p = 0.00096617). Most of the associations between psychosocial work environment factors and the two outcomes, however, were quite modest.

**Table 5:** Multivariate associations between psychosocial work-environment factors and mental health and sleep quality

			**Mental Health**		**Sleep Quality**	
			**Model 1**	**Model 2**	**Model 1**	**Model 2**
**Demographics**						
Women vs. Men			1.47 (0.89–2.41)	**1.88 (1.12–3.15)**	1.12 (0.66–1.90)	1.23 (0.72–2.11)
Age			0.98 (0.97–0.99)	**0.97 (0.96–0.99)**	1.00 (0.99–1.01)	1.00 (0.99–1.01)
**Psychosocial work environment factors**						
Quantitative Demands	Low	Ref	1.00	**1.00**	1.00	1.00
	High		2.75 (1.91–3.97)	**1.99 (1.34–2.95)**	1.54 (1.06–2.23)	1.23 (0.83–1.82)
Work pace	Low	Ref	1.00	**1.00**	1.00	1.00
	High		1.52 (1.17–1.97)	**1.51 (1.13–2.01)**	1.26 (0.68–1.98)	1.17 (0.90–1.52)
Emotional Demands	Low	Ref	1.00	**1.00**	1.00	**1.00**
	High		1.64 (1.19–2.26)	**1.42 (1.00–2.01)**	1.84 (1.35–2.52)	**1.74 (1.25–2.41)**
Influence at work	Low	Ref	1.00	1.00	1.00	1.00
	High		0.75 (0.50–1.14)	0.72 (0.46–1.13)	0.87 (0.60–1.27)	0.90 (0.61–1.29)
Meaning of work	Low	Ref	1.00	1.00	1.00	1.00
	High		0.66 (0.51–0.85)	0.76 (0.56–1.03)	0.89 (0.69–1.14)	0.97 (0.74–1.29)
Involvement in workplace	Low	Ref	1.00	1.00	1.00	1.00
	High		0.57 (0.41–0.80)	0.91 (0.62–1.34)	0.75 (0.54–1.06)	0.97 (0.66–1.41)
Role clarity	Low	Ref	1.00	1.00	1.00	1.00
	High		0.58 (0.45–0.77)	0.83 (0.60–1.15)	0.79 (0.61–1–03)	0.93 (0.68–1.27)
Predictability	Low	Ref	1.00	1.00	1.00	1.00
	High		0.56 (0.43–0.73)	0.80 (0.59–1–10)	0.76 (0.59–0.97)	0.95 (0.70–1.27)
Vertical trust	Low	Ref	1.00	1.00	1.00	1.00
	High		0.55 (0.41–0.73)	0.76 (0.54–1.07)	0.73 (0.56–0.95)	1.01 (0.74–1.38)
Justice and respect	Low	Ref	1.00	1.00	1.00	**1.00**
	High		0.54 (0.41–0.71)	0.82 (0.58–1–14)	0.64 (0.49–0.82)	**0.71 (0.52–0.98)**
Possibilities of development	Low	Ref	1.00	1.00	1.00	1.00
	High		0.74 (0.57–0.96)	0.93 (0.68–1.27)	0.82 (0.63–1.06)	0.89 (0.66–1.18)
Hosmer Lemeshow Goodness of fit				Chi^2^ = 1589		Chi^2^ = 1575
				p = 0.1788		p = 0.284
Nagelkerke Pseudo R^2^				0.07		0.02
Model 1: Adjusted for sex, age, Body Mass Index, Physical Activity, Marital status & Alcohol consumption.						
Model 2: Model 1 adjusted for all other psychosocial work environment indicators.						

Logistic regression. Odds ratios with 95% Confidence Intervals.

Finally, Table 6 shows the results of the multivariate analyses of both physical and psychosocial work environment and self-rated general health. As expected, age is associated with higher odds of bad self-rated general health, but there were no sex differences. None of the physical work environment factors were significantly associated with self-rated general health. However, influence and meaning at work as well as vertical trust and possibilities of development were all associated with self-rated health in the expected direction: higher levels of influence, meaning, trust and possibilities of development all reduced the odds of reporting bad self-rated health. None of these associations had p-values below the Benjamini-Krieger-Yekutieli corrected critical value.

**Table 6:** Multivariate associations between physical and psychosocial work-environment factors and self-rated health

			**Self-rated Health**	
			**Model 1**	**Model 2**
**Demographics**				
Sex	Men	Ref	1.00	1.00
	Women		1.45 (0.89–2.37)	1.55 (0.92–2.62)
Age			1.03 (1.02–1.05)	**1.04 (1.03–1.06)**
**Psychosocial work environment factors**				
Quantitative Demands	Low	Ref	1.00	1.00
	High		1.43 (1.01–2.02)	1.20 (0.82–1.77)
Work pace	Low	Ref	1.00	1.00
	High		0.96 (0.76–1.20)	0.96 (0.74–1.24)
Emotional Demands	Low	Ref	1.00	1.00
	High		1.34 (1.04–1.73)	1.25 (0.94–1.66)
Influence at work	Low	Ref	1.00	**1.00**
	High		0.62 (0.44–0.88)	**0.68 (0.47–0.99)**
Meaning of work	Low	Ref	1.00	**1.00**
	High		0.61 (0.48–0.76)	**0.72 (0.55–0.93)**
Involvement in workplace	Low	Ref	1.00	1.00
	High		0.87 (0.63–1.19)	1.33 (0.92–1.92)
Role clarity	Low	Ref	1.00	1.00
	High		0.65 (0.51–0.83)	0.88 (0.66–1.18)
Predictability	Low	Ref	1.00	1.00
	High		0.73 (0.59–0.92)	1.07 (0.81–1–41)
Vertical trust	Low	Ref	1.00	**1.00**
	High		0.56 (0.45–0.71)	**0.74 (0.55–0.99)**
Justice and respect	Low	Ref	1.00	1.00
	High		0.58 (0.46–0.72)	0.77 (0.57–1–03)
Possibilities of development	Low	Ref	1.00	**1.00**
	High		0.62 (0.49–0.78)	**0.77 (0.59–0.99)**
**Physical work environment factors (daily exposure yes/no)**				
Lift very heavy loads (more than 20 kg)?	No	Ref	1.00	1.00
	Yes		1.13 (0.83–1.53)	0.84 (0.59–1.20)
Lift in an awkward posture?	No	Ref	1.00	1.00
	Yes		1.50 (1.18–1.92)	1.34 (0.96–1.87)
Not enough room around you to perform your work properly?	No	Ref	1.00	1.00
	Yes		1.33 (1.05–1.68)	0.93 (0.66–1.30)
Perform short, but maximal force-exertions?	No	Ref	1.00	1.00
	Yes		1.20 (0.96–1.51)	1.07 (0.80–1.42)
Lift a load that is hard to hold?	No	Ref	1.00	1.00
	Yes		1.38 (1.10–1.73)	1.05 (0.78–1.42)
Make sudden, unexpected movements?	No	Ref	1.00	1.00
	Yes		1.27 (1.01–1.59)	1.08 (0.80–1.45)
Difficulty in exerting enough force because of uncomfortable postures?	No	Ref	1.00	1.00
	Yes		1.27 (1.01–1.60)	0.92 (0.66–1.30)
Not enough room above you to perform your work properly?	No	Ref	1.00	1.00
	Yes		1.32 (1.04–1.67)	1.13 (0.83–1.53)
Too few facilities to lean on during work?	No	Ref	1.00	1.00
	Yes		1.22 (0.95–1.57)	1.01 (0.73–1.39)
Slip or fall during your work?	No	Ref	1.00	1.00
	Yes		1.83 (1.02–3.32)	1.36 (0.70–2.62)
Hosmer Lemeshow Goodness of fit				Chi2 = 1517
				p = 0.242
Nagelkerke Pseudo R2				0.13
Model 1: Adjusted for sex, age, Body Mass Index, Physical Activity, Marital status & Alcohol consumption.				
Model 2: Model 1 adjusted for all other physical and psychosocial work environment indicators.				

Logistic regression. Odds ratios with 95% Confidence Intervals.

## Discussion

This study compares the health status and exposure to psychosocial and physical work environment factors of ambulance personnel with those of the general work force. In addition, analyses of the association between health and work environment factors were carried out to examine which aspects of the general work environment are important for the health status of workers in this occupation.

### Health status

Prior studies have found high prevalences of both musculoskeletal and mental health problems among ambulance personnel [1]. A Japanese study of 1550 paramedics found a twelve month prevalence of neck and shoulder problems around 35%, which is close to our findings, while a low back pain prevalence of 67% was somewhat higher than in our population [15]. However, the results are difficult to compare because we used a stricter criteria for categorising those reporting musculoskeletal pain. More than 85% of the Danish ambulance personnel reported pain in the neck, arm and shoulder as well as low back pain at least once in a 12 month period. Overall, the current study replicates the musculoskeletal problems found in prior research, and the study in addition confirms the earlier findings that ambulance personnel have a higher prevalence of musculoskeletal pain when compared to the general work force [25]. This was not the case for mental health issues, where no significant differences between ambulance personnel and the general work force were observed. However, it is difficult to compare our result directly to earlier studies, because the measures used in prior research are either not described or not the same as in this study [10-13]. Despite the higher prevalence of musculoskeletal pain among ambulance personnel, a significantly larger proportion still self-rates their health as better than the self-ratings of the general work force. Although we do not know the exact reason for this, there are several plausible explanations: First of all, health selection may be at work: the most fragile ambulance workers health-wise may have changed job function internally in the organisation resulting only in the most healthy ambulance personnel remaining. Second, the level of physical activity among the ambulance personnel is substantially higher than that of the general work force, which could also help explain the better health status of this group (results not shown). Only prospective analyses of the next round of data in our study will be able to shed more light on this difference.

### Physical work environment

Despite the known high levels of exposure to physical work demands required in ambulance work, only a few quality studies on this topic have been conducted on the issue of musculoskeletal disorders. This study shows that ambulance personnel are exposed to a wide variety of factors in the physical work environment including lifting in awkward postures, lifting loads that are hard to hold, performing short, maximal force-exertions and having too few facilities to lean on during work. Of the 10 factors, three were associated with higher levels of musculoskeletal pain after adjusting mutually for each other and for all the other confounder variables. Performing short maximal-force exertions was the only factor associated with both types of musculoskeletal pain. Although none of the estimates had p-values below the Benjamini-Krieger-Yekutieli corrected critical value, we wonder whether this may be too harsh a criterion on which to judge these estimates. Because of the dynamic character of the work, none of the exposures can be seen completely in isolation and for this reason, adjusting for all the other physical work environment factors at the same time may result in over adjustment.

A Swedish study identifying physical risk factors as forward bending and twisting postures corresponded to our findings, while they in contrast also found handling of heavy tasks of significant importance [14]. In multivariate analyses, they found that the highest risks for pain conditions leading to limitation in physical activity for the female personnel were work in awkward postures, both in relation to neck-shoulder (OR 3.0), and low back pain (OR 1.9).

Most of the research on musculoskeletal risk factors focuses on heavy lifting, bending, etc. This study points to the need for more detailed knowledge of exposures, such as short maximal force –exertions and brief awkward postures. However, our findings on the ergonomic factors are solely based on questionnaire-derived exposure data, which have been shown to be vulnerable to misclassification due to musculoskeletal complaints, and interpretations must take this into account [26]. Exposure assessment by standardised observation and direct measurements would be desirable, but because of the unpredictable nature of the work-environment, this is not easily carried out.

Despite the finding that participants experienced more upper extremity pain and low back pain than the core work force, this cross-sectional study cannot elucidate causal relationships. In addition, only one of the specific items in the physical work environment was associated with both pain measures. This lack of consistency and the fact that the p-values did not reach below the critical values corrected for false discovery rates should stop us from drawing conclusions that are too unequivocal on this. The results could indicate the importance of different physical exposures in the occurrence of pain, but they might also reflect recall bias or Type I errors. Later prospective data will inform us of this in more detail.

### Psychosocial work environment

The current study points to two aspects of the psychosocial work environment where ambulance personnel experience more demanding work conditions than the general work force. Influence at work is substantially lower than in the rest of the work force, while the level of emotional demands is substantially higher. It is hardly surprising that emotional demands are higher and that the influence at work is smaller given the nature of most of the work tasks and the need for urgency related to them. However, the nature of the work serves as a barrier to improving the psychosocial work environment for this particular occupation.

On the other hand, the levels of meaning of work and commitment to the workplace reported by the ambulance personnel show that the perception of the work as very important and as having an intrinsic meaning may serve as a buffer against the potential harms of an excessive level of emotional demands. This was the case in a Scottish study where those scoring high on subscales of commitment and control had significantly fewer burn-out symptoms [27]. Other studies have found that social support serves as a buffer for mental health problems [28], and social environment and personal resources have been suggested as important mediating or modifying factors [3]. In our study the multivariate analyses show that higher levels of emotional demands lead to higher odds of bad mental health and bad sleep quality. Prospective analyses based on this cohort will show whether these findings can be replicated.

The study by Aasa et al. resembles the present one in the purpose of surveying psychosocial factors [13]. They had a representative sample of 1287 ambulance technicians and nurses and a response rate of 79%, but unfortunately no comparison group. However, overall scores of psychological demand and decision latitude could be compared to some other Swedish occupational groups and was lower than for physicians and air traffic controllers. They found in multivariate analyses that psychological demands during emergency call-outs and worry about work conditions (worry about making mistakes, being injured at work, or being subjected to threats or violence) were significantly associated with sleeping problems as well as somatic stress symptoms. Even if this is not directly comparable, such worries may be seen as one aspect of the items tapping into emotional demands that were used in our study.

A Dutch follow-up study found that ambulance personnel scored significantly higher on subscales measuring psychological emotional demands [9], which is in accordance with our findings. They also found that although acute stressors were related to complaints like fatigue, burnout and PTSD symptoms, they did not predict long term health symptoms [9]. Unfortunately, the response rate at follow up was only 31%.

Many studies report significant associations between job stressors and mental health problems, but without controlling for explanatory variables such as personality factors, coping and social support, reliable causal inference cannot be made. The main findings in the present study are the significant effect of emotional demands on mental health and sleep quality. Our study should contribute to the evidence base in the field of psychosocial work environment for workers employed in emergency rescue service, given our large representative study population, relevant comparison group, and control for confounders.

### Limitations of the study

The main weakness of the current study is the cross-sectional design. One crucial problem for the analyses is the possibility that health-selection is at work: from our qualitative studies of the company, we know that it has been a practice to transfer personnel with lower work ability to job functions other than ambulance driving. This could have the effect of causing the results of this study to underestimate the true association between the work environment exposures and health. The study, however, is designed as a prospective cohort study and future data will make it more appropriate for us to examine possible causal mechanisms linking different work-environment factors and health status among ambulance personnel more precisely.

Nearly all studies of this occupation are of European or Anglo-Saxon origin, and it should be presumed that national differences in organisation, education, urban/rural exposure and many other factors reduce the potential for making generalisations [29].

The differences in psychosocial work-environment factors were small compared to the workforce in general. The lack of contrast was also revealed as rather small associations in the multivariate analysis (Table 5). It looks as if ambulance personnel had higher emotional demands, which seem explainable by the nature of their job, but whether this could cause mental health problems at a later time cannot be answered by the current data. There was a small, although significant, effect of emotional demands on mental health in the multivariate analysis, but at the same time ambulance personnel were in general at lower risk of poor mental health, and we can make no conclusion about this in this cross-sectional comparison.

## Conclusions

### Implications for policy and practice

Our study shows that musculoskeletal problems should be a focus area in improving the work environment for ambulance personnel. Procedures and guidelines for safe lifting are important and are often specified, but adherence to them can be difficult under real life conditions. Therefore, attention should also be paid to the availability and development of new facilities for lifting heavy and difficult loads that would minimize manual lifting in awkward postures and especially the need to perform short maximum force exertions.

The nature of emergency work makes it difficult to change the exposure to some of the psychosocial work environment factors, such as unpredictability and high emotional demands. To counteract the potential harmful influence of these factors, focus should be on ensuring the presence of positive organizational support mechanisms, such as influence, social support, organizational justice, and trust, which might serve as protective buffers against mental health problems.

## Competing interests

All authors declare that they have no competing interests.

## Authors’ contributions

CH, KN and MK jointly conceived the idea for the paper. CH and JA performed the statistical analyses. All authors interpreted the data. CH, KR, KN and MK jointly drafted the manuscript. JA critically revised the paper. CH will act as guarantor for the paper. All authors approved the final manuscript.

## Pre-publication history

The pre-publication history for this paper can be accessed here:

http://www.biomedcentral.com/1471-2458/12/534/prepub
